# Supplementary material for: Assessment of the Effectiveness of a Computerised Decision-Support Tool for Health Professionals for the Prevention and Treatment of Childhood Obesity. Results from a Randomised Controlled Trial
Source: Nutrients. 2019 Mar 26;11(3):706. doi: 10.3390/nu11030706 (PMC6471646; doi:10.3390/nu11030706)
Supplement: Supplementary file 1 [file nutrients-11-00706-s001.zip › nutrients-456694-SI/Supplementary Material/Supplementary Figure S1. Decision tree algorithms.pdf]

Evaluation of children’s weight status

Step 1

Body Weight (Kg) / Heaight^2 (m^2) = BMI (Kg/m^2)

Evaluation of child’s weight status based on the appropriate BMI-for-age growth curve

Normal-weight (BMI <85° percentile)

Continue with the decision tree algorithm for normal-weight children

Overweight (BMI ≥85° to < 95° percentile)

Continue with the decision tree algorithm for overweight children

Obese (BMI ≥ 95° percentile)

Continue with the decision tree algorithm for obese children

Decision tree algorithm for normal-weight children

Step 2 (Continue from Step 1 for normal-weight children)

Evaluation of the likelihood for the future manifestation of obesity based on the CORE-index score

Lower likelihood (CORE index score < 4)

Provide general age-specific recommendations for healthy growth and nourishment

Recommendation 1

Higher likelihood (CORE index score ≥ 4)

Continue with the decision tree algorithm for normal-weight children with a higher likelihood for the future manifestation of obesity

Decision tree algorithm for normal-weight children with a higher likelihood for the future manifestation of obesity

Step 3 (Continue from Step 2)

Normal-weight children with a higher likelihood for the future manifestation of obesity

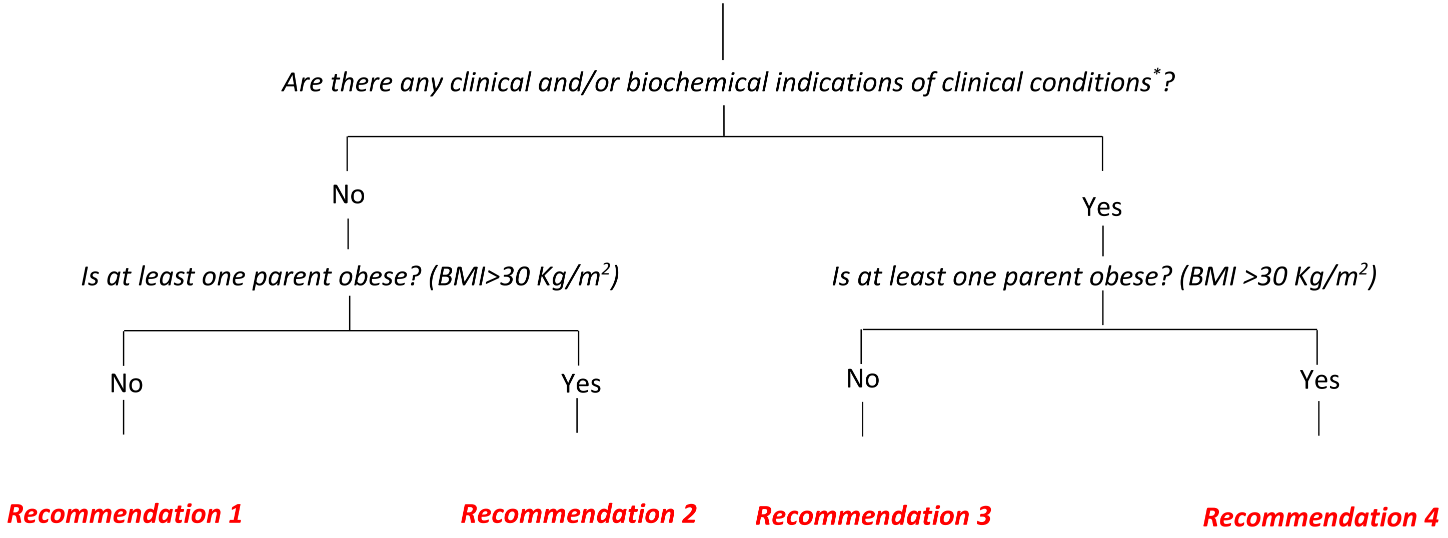

*Clinical conditions include metabolic disorders, such as hyperglycaemia, insulin resistance, dyslipidaemias, hypertension based on indications stemming from the clinical examination of the child (e.g. increased blood pressure levels etc.) and on the elevated levels of biochemical indices available to the health professionals or to the family.

Decision tree algorithm for overweight children

Step 4 (Continue from Step 1 for overweight children)

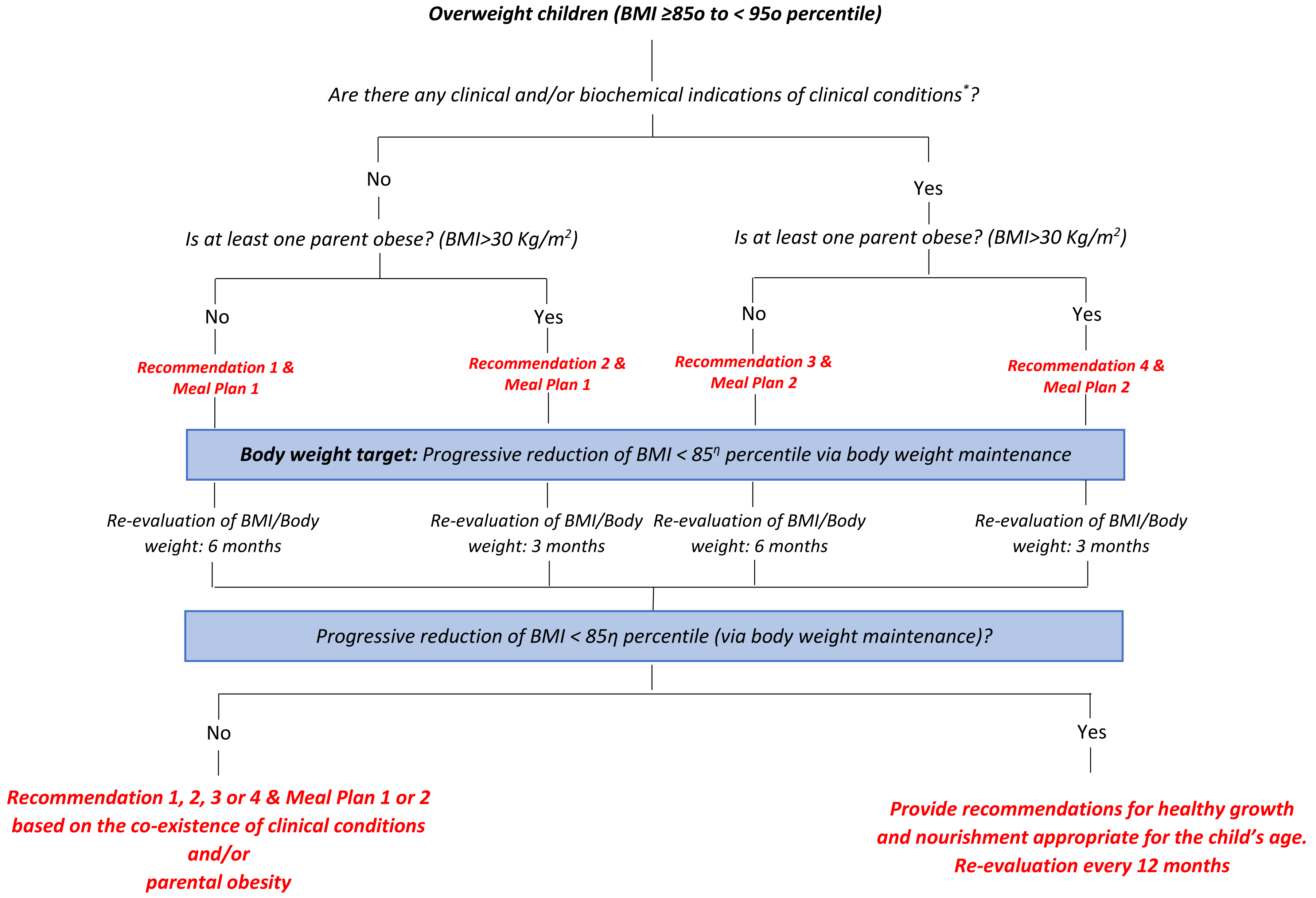

*Clinical conditions include metabolic disorders, such as hyperglycaemia, insulin resistance, dyslipidaemias, hypertension based on indications stemming from the clinical examination of the child (e.g. increased blood pressure levels etc.) and on the elevated levels of biochemical indices available to the health professionals or to the family.

Decision tree algorithm for obese children

Step 5 (Continue from Step 1 for obese children)

Obese children (BMI ≥ 95th percentile)

Are there any clinical and/or biochemical indications of clinical conditions?*

- **No** → *Is at least one parent obese? (BMI>30 Kg/m^2)*
  - **No** →
    - **2-5 years old:** **Recommendation 1 & Meal Plan 1**
    - **6-15 years old:** (none listed)
  - **Yes** →
    - **2-5 years old:** **Recommendation 2 & Meal Plan 1**
    - **6-15 years old:** **Recommendation 2 & Meal Plan 3**
- **Yes** → *Is at least one parent obese? (BMI>30 Kg/m^2)*
  - **No** →
    - **2-5 years old:** **Recommendation 3 & Meal Plan 2**
    - **6-15 years old:** **Recommendation 3 & Meal Plan 4**
  - **Yes** →
    - **2-5 years old:** **Recommendation 4 & Meal Plan 2**
    - **6-15 years old:** **Recommendation 4 & Meal Plan 4**

Body weight target: *Progressive reduction of BMI < 85η percentile, via body weight maintenance in 2-5 year-old children with BMI ≥ 95th percentile and via body weight maintenance or mild weight loss for 6-15 year-old children/adolescents with BMI between > 95th percentile*

- Recommendation 1 & Meal Plan 1: *Re-evaluation of BMI/Body weight: 6 months*
- Recommendation 2 & Meal Plan 1 / 3: *Re-evaluation of BMI/Body weight: 3 months*
- Recommendation 3 & Meal Plan 2 / 4: *Re-evaluation of BMI/Body weight: 6 months*
- Recommendation 4 & Meal Plan 2 / 4: *Re-evaluation of BMI/Body weight: 6 months*

Progressive reduction of BMI < 85η percentile (via body weight maintenance)?

- **No** → ***Recommendation 1, 2, 3 or 4 & Meal Plan 1, 2, 3 or 4 based on the co-existence of clinical conditions and/or parental obesity***
- **Yes** → ***Provide recommendations for healthy growth and nourishment appropriate for the child's age. Re-evaluation every 12 months***

*Clinical conditions include metabolic disorders, such as hyperglycaemia, insulin resistance, dyslipidaemias, hypertension based on indications stemming from the clinical examination of the child (e.g. increased blood pressure levels etc.) and on the elevated levels of biochemical indices available to the health professionals or to the family.
