# Supplementary material for: Assessment of the Effectiveness of a Computerised Decision-Support Tool for Health Professionals for the Prevention and Treatment of Childhood Obesity. Results from a Randomised Controlled Trial
Source: Nutrients. 2019 Mar 26;11(3):706. doi: 10.3390/nu11030706 (PMC6471646; doi:10.3390/nu11030706)
Supplement: Supplementary file 1 [file nutrients-11-00706-s001.zip › nutrients-456694-SI/Supplementary Material/Supplementary Table S1 Lifestyle recommendations.pdf]

children 9—12 years old

Tips towards a healthy lifestyle

The role of a healthy nutrition in academic and athletic performance and towards a healthy child growth

- Adopting a healthy nutrition is very important for the children's physical and mental health.
- A healthy nutrition provides children with the energy and nutrients required daily, particularly when high activity levels are very common at this age.
- Studies have shown that children consuming healthy foods have better memory, concentration, academic and athletic performance, as well as a more strengthened immune system than most children.
- Dietary habits acquired during childhood are the cornerstone for maintaining health and well-being during adulthood.

Eating healthy snacks:

- Children should consume 3 main meals daily at regular intervals.
- Choose to offer at least 1 small snack, low in fat, salt and sugar (e.g. toasted bread with low fat cheese and vegetables).
- A snack should contain healthy choices from at least 2 food groups (e.g. yogurt with fruits).
- Always have healthy homemade snacks at the ready for your children to prevent them from choosing a less healthy snack.
- Offer water or milk instead of pre-packed fruit juices. If you choose to offer juice, opt for a 100% fresh juice.
- Avoid processed snacks (e.g. potato chips, cookies, etc.).
- Offer different food choices to prevent children from getting bored.
- Do not use sweets as rewards.

Ideal snack options:

- Sandwich or toasted bread with vegetables (tomato, lettuce, cabbage-carrot) and cheese or cooked (boiled or roasted) chicken/turkey or egg
- Fruits and/or vegetables (e.g. cucumber and carrot sticks)
- Yogurt with fruits or raisins
- Unsalted nuts (for older children)

Physical activity guidelines for children

Children at this age are recommended to:

- Accumulate at least ***60 minutes*** of moderate - to vigorous -intensity physical activity ***daily*** either continuously or in multiple shorter bouts of at least 10 minutes spread throughout the day.
- Participate in vigorous-intensity activities, including those that strengthen muscle and bone, at ***least 3 times per week***.
- Physical activities include either structured (e.g. sports) or unstructured activities (e.g. walking, cycling, swimming)
- For children under the age of 10, enjoyable and safe non-competitive activities should be preferred.

Examples of moderate - to vigorous-intensity physical activities:

- Cycling
- Playground activities
- Swimming
- Playing tag
- Brisk walking/jogging
- Ice-skating or road-skating
- Dancing

Examples of vigorous-intensity activities, that strengthen muscle and bone:

- Climbing walls, trees, ropes
- Use of ladder
- Hopscotch
- Rope jumping
- Gymnastics, basketball, volleyball
- Yoga
- Martial arts
- Running

Sleep habits

Children aged 9-12 years need 9-12 hours of sleep at a daily basis. To ensure optimum sleep quality:

- Turn off the TV and any other screen* at least 30 minutes before your child falls to sleep
- Remove the TV or any other screen from children's bedroom.
- Make sure the bedroom is as neat as possible .
- Maintain a steady sleep schedule and let your child become more responsible as he/she grows older.

**Screen is defined as: TV, PC, smartphones, tablets, videogames, etc.*

Sedentary activities

For children of this age it is recommended to:

- Reduce recreational screen* time to no more than 1-**2 hours per day**.
- Limit sedentary (motorized) transport.

How to limit sedentary time:

- Set specific hours throughout the day that children will spend on TV or other screen.
- Remove the TV or any other screen from children's bedroom.
- Instead of driving, walk to school or to extra curriculum activities.
- Take your pet for a walk along with your child.
- Get up along with your child and move indoors for 2-5 minutes after prolonged sitting time periods (duration ≥1 hour).
- Do not use screen time as punishment or reward (e.g. "You will watch TV if you do your homework".).

**Screen is defined as: TV, PC, smartphones, tablets, videogames, etc.*

Dietary Recommendations for 9-12 years old children

Food Group	Recommended servings	Size of servings
Vegeta-bles	2-3 servings/ day	1 cup. cooked or raw vegetables,<br>2 cups raw green vegetables<br>1 medium cucumber,<br>1 big tomato or 1 cup grated tomato<br>2 medians carrots
Fruit	2-3 servings/ day	1 medium fruit (e.g. 1 apple),<br>2 small fruits (e.g. 2 tangerines),<br>30 small grapes,<br>1 slice of watermelon or melon,<br>4 dried fruits (e.g. plums),<br>1,5 tablespoon of raisins,<br>8 strawberries<br>15 cherries<br>½ cup (125 ml) fresh juice
Milk and dairy products	3-4 servings/ day	1 cup (250 ml) milk or ½ cup (125 mL) evaporated milk<br>1 cup (200 gr) yogurt<br>30gr hard cheese<br>2 tablespoons of soft cheese
Cereal (bread, rice, pas-ta) & po-tato	5-6 servings/ day	1 slice of bread (30 gr) or 2 small rusks or 1 medium rusk,<br>½ cup cereals<br>½ round sesame bread,<br>½ cup of cooked pasta or rice,
Legumes	At least 3 servings/ week	90-120g cooked and drained leg-umes
White and red meat	2-3 servings/ week	90-120g cooked meat
Fish and Seafood	2-3 servings/ week	120-150gr cooked fish without bones or cooked seafood
Eggs	4-7 / week	1 egg
Added fat and oils, olives and nuts	3-4 servings/ week	1 tablespoon of olive oil or other vegetable oil or butter or marga-rine, 1 handful of nuts (e.g. 18 al-monds, 6 whole walnuts, 3 table-spoons of sun flower seeds),<br>10-12 olives<br>1,5 tablespoon of tahini

Indicative Diet Meal Plan for children 9-12 years old

Breakfast.

- 1 cup milk (250ml) 1.5% fat or 1 cup yogurt 2% fat
- 1 slice of bread (30g) or 2 rusks or ½ cup of cereals

Morning Snack:

- 30g cheese (low fat) or cheese & turkey sliced or 1 egg
- 2 slices of bread (60g) or 4 rusks
- Vegetables (e.g. tomato, lettuce, cucumber)
- 1 fruit

Lunch:

- 80g red meat or chicken or fish
- Plenty of vegetables
- 3 teaspoons of olive oil
- 2/3 cup pasta or rice or 1 median potato or 1 cup legumes
- 1 slice of bread(30g) or 2 rusks
- 1 fruit

Afternoon Snack:

- 1 cup milk (250ml) 1.5% fat or 1 cup yogurt 2% fat
- 1 slice of bread (30g) or 2 rusks

Dinner:

- 55g red meat or chicken or fish
- Plenty of vegetables
- 3 teaspoons of olive oil
- ½ cup pasta or rice or 1 median potato or ¾ cup legumes
- 0,5 slice of bread (15g) or 1 rusk
- 1 fruit

Before Bedtime:

- 1 cup milk (250ml) 1.5% fat or 1 cup yogurt 2% fat

Source: Linou, A., et al. *National dietary guidelines for infants, children and adolescents*. National Die-tary Guidelines "Eu Dia...Trofin", pub. Institute of Preventive Medicine Environmental and Occupational Health Prolepsis. 2014: Labrakis Press S.A.

Ten steps to Healthy Eating for children and adolescents

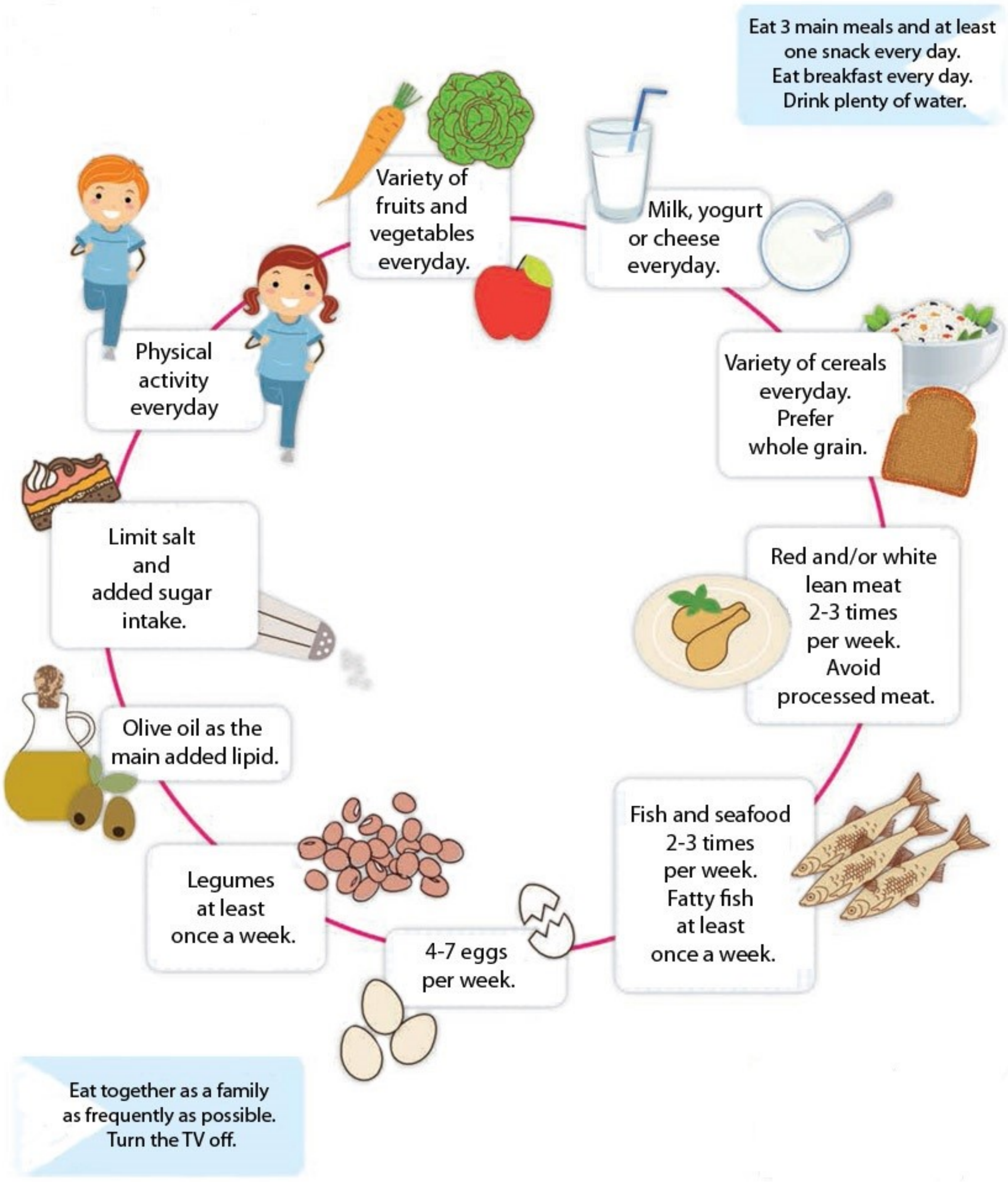

Source: Linou, A., et al. *National dietary guidelines for infants, children and adolescents*. National Dietary Guidelines "Eu Dia...Trofin", pub. Institute of Preventive Medicine Environmental and Occupational Health Prolepsis. 2014: Labrakis Press S.A.

children 9—12 years old

Tips towards a healthy lifestyle

The role of healthy nutrition

- Adopting a healthy nutrition is very important not only for the children's physical and mental health, as well as for the whole family's.
- Healthy dietary habits provide us with the energy and nutrients required daily at every age.
- Your child's nutrition at this stage contributes actively to his growth, since by the age of 10, a child's weight is increased by 3 kg per year and its height by about 6 cm, up to the pre-puberty period.
- Studies have shown that children consuming healthy foods have better memory, concentration, academic and athletic performance, as well as a more strengthened immune system than most children
- Dietary habits acquired during childhood are the cornerstone for maintaining health and well-being during adulthood.

Dietary tips for the whole family

- All family members should consume 3 main meals daily at regular intervals. Children should eat at least 1 small snack (e.g. 1 cup of yogurt with fruits), while adults can also eat snacks between main meals.
- Be a role model for your child by eating a balanced breakfast that includes foods from at least 3 food groups (dairy, fruit, cereal).
- Snacks may contain food choices from 2 or more food groups (e.g. round sesame bread and a fruit).
- Always have healthy homemade snacks (e.g. cut fruits, vegetables) at the ready to prevent them from choosing a less healthy snack.
- Limit the intake of fat, salt and added sugars.
- Make sure to eat as a whole family, without interference and turn the meals into a pleasant experience for everyone .
- Encourage children to try new foods by repeated exposure .
- Include children at all stages of a meal preparation from an early age.
- Serve the same meal to all family members. Avoid preparing something special for children.
- Limit take-out to a minimum of 1 per week
- Do not use sweets as rewards.

Physical activity guidelines for children

Children at this age are recommended to:

- Accumulate at least ***60 minutes*** of moderate - to vigorous-intensity physical activity ***daily*** either continuously or in multiple shorter bouts of at least 10 minutes spread throughout the day.
- Participate in vigorous-intensity activities, including those that strengthen muscle and bone, at ***least 3 times per week***.
- Physical activities include either structured (e.g. sports) or unstructured activities (e.g. walking, cycling, swimming)
- For children under the age of 10, enjoyable and safe non-competitive activities should be preferred.

Examples of moderate - to vigorous-intensity physical activities:
- Cycling
- Playground activities
- Swimming
- Playing tag
- Brisk walking/jogging
- Ice-skating or road-skating
- Dancing

Examples of vigorous-intensity activities, that strengthen muscle and bone:
- Climbing walls, trees, ropes
- Use of ladder
- Hopscotch
- Rope jumping
- Gymnastics, basketball, volleyball
- Yoga
- Martial arts
- Running

Examples of moderate intensity physical activities for adults:
- Brisk walking
- Dancing
- Gardening
- Household activities
- Active play with children
- Walk with pet
- Cycling <15km/hour
- Carrying load <20kg

Examples of vigorous intensity physical activities for adults:
- Running
- Walking on a slope
- Cycling >15km/hour
- Swimming
- Aerobics
- Competitive sports (basketball, football, etc.)
- Carrying load >20kg

Physical activity guidelines for adults

For adults aged 18-64 years it is recommended to:

- Do **at least 150 minutes** of moderate-intensity aerobic physical activity **throughout the week (or 30 minutes per day)** or do **at least 75 minutes** of vigorous-intensity aerobic physical activity **throughout the week (or 15 minutes per day** either continuously or in multiple shorter bouts of at least 10 minutes spread throughout the day.
- Participate in muscle-strengthening activities **on 2 or more days a week.**

Sedentary activities for children

For children of this age it is recommended to:

- Reduce recreational screen* time to **no more than 1-2 hours per day**.
- Limit sedentary (motorized) transport.

Sedentary activities for adults

For adults it is recommended to:

- Limit sedentary habits as much as possible and make regular movement breaks.

How to limit sedentary time:

- Set specific hours throughout the day that family members will spend on TV or other screen
- Remove the TV or any other screen from family members' bedroom
- Instead of driving, use public transportation and get off the bus/metro 2-3 stops before your final destination.
- Instead of driving, walk to school or to extra curriculum activities along with your child.
- Take your pet for a walk along with your child.
- Get up along with your child and move indoors for 2-5 minutes after prolonged sitting time periods (duration ≥1 hour).
- Do not use screen time as punishment or reward (e.g. "You will watch TV if you do your homework".).

Sleep habits for children

Children aged 9-12 years need 9-12 hours of sleep at a daily basis. To ensure optimum sleep quality:

- Turn off the TV and any other screen* at least 30 minutes before your child falls to sleep
- Remove the TV or any other screen from children's bedroom.
- Make sure the bedroom is as neat as possible .
- Maintain a steady sleep schedule and let your child become more responsible as he/she grows older.

** Screen is defined as: TV, PC, smartphones, tablets, video-games, etc.*

Dietary Recommendations for 9-12 years old children

Food Group	Recommended servings	Size of servings
Vegetables	2-3 servings/ day	1 cup. cooked or raw vegetables,<br>2 cups raw green vegetables<br>1 medium cucumber,<br>1 big tomato or 1 cup grated tomato<br>2 medians carrots
Fruit	2-3 servings/ day	1 medium fruit (e.g. 1 apple),<br>2 small fruits (e.g. 2 tangerines),<br>30 small grapes,<br>1 slice of watermelon or melon,<br>4 dried fruits (e.g. plums),<br>1,5 tablespoon of raisins,<br>8 strawberries<br>15 cherries<br>½ cup (125 ml) fresh juice
Milk and dairy products	3-4 servings/ day	1 cup (250 ml) milk or ½ cup (125 mL) evaporated milk<br>1 cup (200 gr) yogurt<br>30gr hard cheese<br>2 tablespoons of soft cheese
Cereal (bread, rice, pasta) & potato	5-6 servings/ day	1 slice of bread (30 gr) or 2 small rusks or 1 medium rusk,<br>½ cup cereals<br>½ round sesame bread,<br>½ cup of cooked pasta or rice,<br>1 medium cooked potato
Legumes	At least 3 servings/ week	90-120g cooked and drained legumes
White and red meat	2-3 servings/ week	90-120g cooked meat
Fish and Seafood	2-3 servings/ week	120-150gr cooked fish without bones or cooked seafood
Eggs	4-7 / week	1 egg
Added fat and oils, olives and nuts	3-4 servings/ week	1 tablespoon of olive oil or other vegetable oil or butter or margarine, 1 handful of nuts (e.g. 18 almonds, 6 whole walnuts, 3 tablespoons of sun flower seeds),<br>10-12 olives<br>1,5 tablespoon of tahini

Indicative Diet Meal Plan for children 9-12 years old

Breakfast.

- 1 cup milk (250ml) 1.5% fat or 1 cup yogurt 2% fat
- 1 slice of bread (30g) or 2 rusks or ½ cup of cereals

Morning Snack:

- 30g cheese (low fat) or cheese & turkey sliced or 1 egg
- 2 slices of bread (60g) or 4 rusks
- Vegetables (e.g. tomato, lettuce, cucumber)
- 1 fruit

Lunch:

- 80g red meat or chicken or fish
- Plenty of vegetables
- 3 teaspoons of olive oil
- 2/3 cup pasta or rice or 1 median potato or 1 cup legumes
- 1 slice of bread(30g) or 2 rusks
- 1 fruit

Afternoon Snack:

- 1 cup milk (250ml) 1.5% fat or 1 cup yogurt 2% fat
- 1 slice of bread (30g) or 2 rusks

Dinner:

- 55g red meat or chicken or fish
- Plenty of vegetables
- 3 teaspoons of olive oil
- ½ cup pasta or rice or 1 median potato or ¾ cup legumes
- 0,5 slice of bread (15g) or 1 rusk
- 1 fruit

Before Bedtime:

- 1 cup milk (250ml) 1.5% fat or 1 cup yogurt 2% fat

__Source:__ Linou, A., et al. *National dietary guidelines for infants, children and adolescents*. National Dietary Guidelines "Eu Dia...Trofin", pub. Institute of Preventive Medicine Environmental and Occupational Health Prolepsis. 2014: Labrakis Press S.A.

Dietary Recommendations for Adults

Food Group	Recommended servings	Size of servings
**Vegetables**	4 servings/ day	1 cup. cooked or raw vegetables,<br>2 cups raw green vegetables<br>1 medium cucumber,<br>1 big tomato or 1 cup grated tomato<br>2 medians carrots
**Fruit**	3 servings/ day	1 medium fruit (e.g. 1 apple),<br>2 small fruits (e.g. 2 tangerines),<br>30 small grapes,<br>1 slice of watermelon or melon,<br>4 dried fruits (e.g. plums),<br>1,5 tablespoon of raisins,<br>8 strawberries<br>15 cherries<br>½ cup (125 ml) fresh juice
**Milk and dairy products**	2 servings/ day	1 cup (250 ml) milk or ½ cup (125 mL) evaporated milk<br>1 cup (200 gr) yogurt<br>30gr hard cheese<br>2 tablespoons of soft cheese
**Cereal (bread, rice, pasta) & potato**	5-8 servings / day of which potato consumption is about 3 servings per week	1 slice of bread (30 gr) or 2 small rusks or 1 medium rusk,<br>½ cup cereals<br>½ round sesame bread,<br>½ cup of cooked pasta or rice,<br>1 medium cooked potato
**Legumes**	At least 3 servings/ week	150–200g cooked and drained legumes (1 cup)
**Red meat**	1 servings/ week	120-150g red cooked meat
**White meat**	1-2 servings/ week	120-150g white cooked meat
**Fish and Seafood**	2-3 servings /week, at least half of the servings are oily fish (eg, sardine, anchovy, coli, zaranana), which are rich in omega-3 fat.	150g of cooked fish or seafood (eg 1 medium sea bream, 10-12 anchovies, 10-12 sardines, 15 medium shrimps, 12-14 big mussels)
**Eggs**	3-4 servings/ week	1 egg
**Added fat and oils, olives and nuts**	4-5 servings/ day (consume olive oil as the first choice of added oil in cooking and salad)	1 tablespoon of olive oil or other vegetable oil or butter or margarine, 1 handful of nuts (e.g. 18 almonds, 6 whole walnuts, 3 tablespoons of sun flower seeds),<br>10-12 olives<br>1,5 tablespoon of tahini

Source: Linou, A., et al. *National dietary guidelines for infants, children and adolescents*. National Dietary Guidelines "Eu Dia...Trofin", pub. Institute of Preventive Medicine Environmental and Occupational Health Prolepsis. 2014: Labrakis Press S.A.

Indicative Diet Meal Plan for Adults

Breakfast:
- 1 cup milk (240ml) 1.5% fat or 1 cup yogurt 2% fat
- 1 cup of cereals or 4 rusks or 2 slices of bread

Lunch:
- 150g fish or chicken or turkey or beef or pork
- 1 cup of rice or pasta or 2 small potatoes or 2 slices of bread
- 2 cups of raw vegetables or 1 cup of boiled vegetables
- 1 tablespoon of olive oil
- 1 slice of bread
- 1 fruit

Or

- 1 cup legumes (e.g. lentils, chickpeas, bean soup, giants beans, split peas) with 1 tablespoon of olive oil / serving
- 60g cheese or 30g cheese with 1 egg
- 5 big or 10 small olives
- 1 slice of bread
- 1 fruit

Or

- 1 cup of cooked vegetables (π.χ. okra, artichokes, eggplant, green beans) with ½ cup of potatoes (or 1 small potato) with 1 tablespoon of olive oil
- 90g fish or 90g chicken or 60g cheese and 1 egg
- 2 slices of bread
- 1 fruit

Dinner:
- 120g chicken or 120g tuna or 120g cheese or 2 eggs
- 1 cup of rice or pasta or 2 small potatoes or 2 slices of bread
- 2 cups of raw vegetables or 1 cup of boiled vegetables
- 1 tablespoon of olive oil
- 1 fruit

Or

- 2 slices of bread, 2 slices of cheese (60g) and 2 slices of turkey (60g)
- 2 cups of raw vegetables or 1 cup of boiled vegetables
- 1 tablespoon of olive oil
- 1 fruit

Snack (Consumed during the day) :
- 1 fruit
- 4 crackers ή 1 sesame round bread
- 1 cup milk (240ml) 1.5% fat or 1 cup yogurt 2% fat

Ten steps to Healthy Eating for children and adolescents

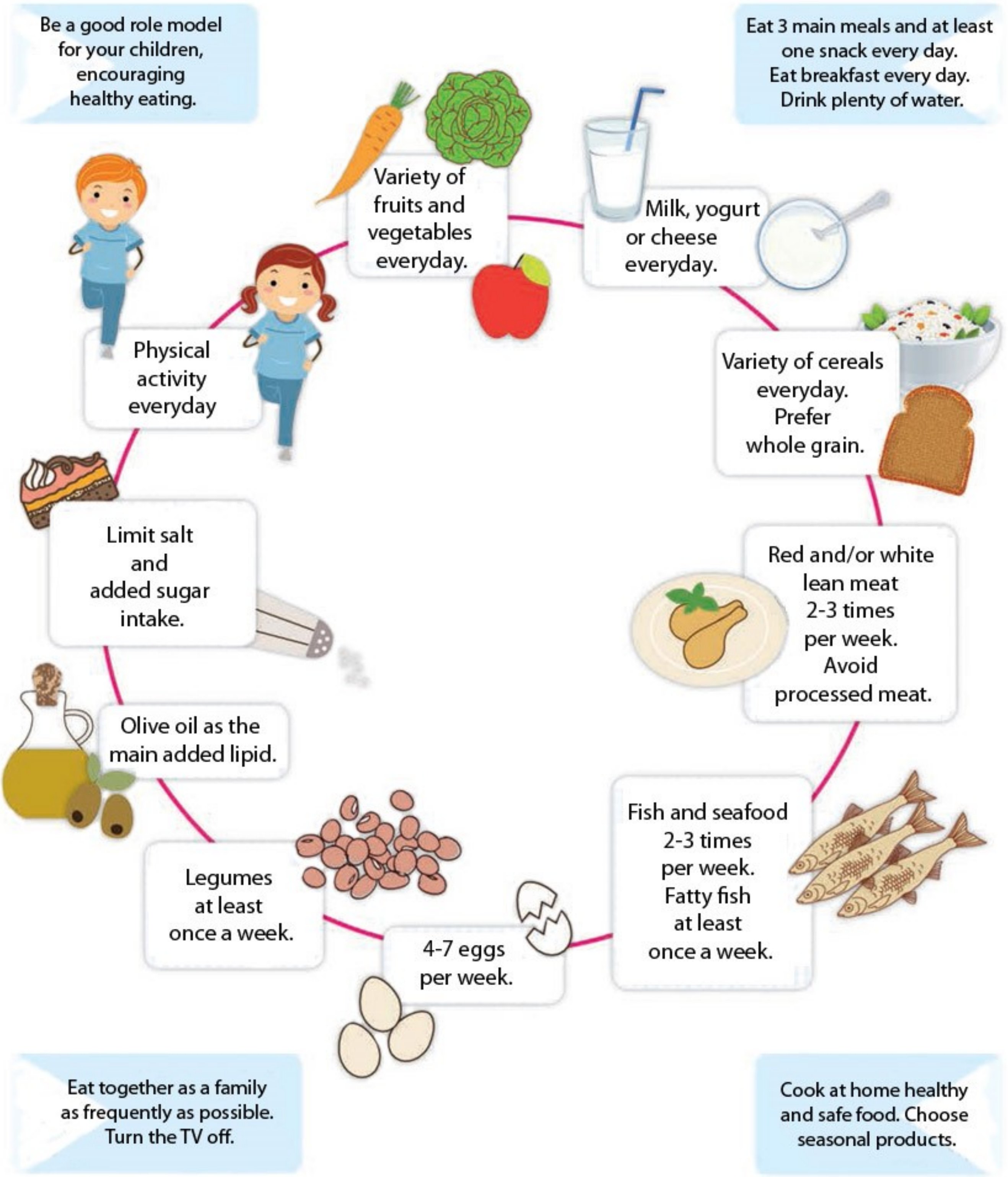

Source: Linou, A., et al. *National dietary guidelines for infants, children and adolescents*. National Dietary Guidelines "Eu Dia...Trofin", pub. Institute of Preventive Medicine Environmental and Occupational Health Prolepsis. 2014: Labrakis Press S.A.

children 9—12 years old

Tips towards a healthy lifestyle

The role of a healthy nutrition in academic and athletic performance and towards a healthy child growth

- Adopting a healthy nutrition is very important for the children's physical and mental health.
- A healthy nutrition provides children with the energy and nutrients required daily, particularly when high activity levels are very common at this age.
- Studies have shown that children consuming healthy foods have better memory, concentration, academic and athletic performance, as well as a more strengthened immune system than most children.
- Dietary habits acquired during childhood are the cornerstone for maintaining health and well-being during adulthood.

Eating healthy snacks:

- Children should consume 3 main meals daily at regular intervals.
- Choose to offer at least 1 small snack, low in fat, salt and sugar (e.g. toasted bread with low fat cheese and vegetables).
- A snack should contain healthy choices from at least 2 food groups (e.g. yogurt with fruits).
- Always have healthy homemade snacks at the ready for your children to prevent them from choosing a less healthy snack.
- Offer water or milk instead of pre-packed fruit juices. If you choose to offer juice, opt for a 100% fresh juice.
- Avoid processed snacks (e.g. potato chips, cookies, etc.).
- Offer different food choices to prevent children from getting bored.
- Do not use sweets as rewards.

Ideal snack options:

- Sandwich or toasted bread with vegetables (tomato, lettuce, cabbage-carrot) and cheese or cooked (boiled or roasted) chicken/turkey or egg
- Fruits and/or vegetables (e.g. cucumber and carrot sticks)
- Yogurt with fruits or raisins

Physical activity guidelines for children

Children at this age are recommended to:

- Accumulate at least ***60 minutes*** of moderate - to vigorous-intensity physical activity ***daily*** either continuously or in multiple shorter bouts of at least 10 minutes spread throughout the day.
- Participate in vigorous-intensity activities, including those that strengthen muscle and bone, at ***least 3 times per week***.
- Physical activities include either structured (e.g. sports) or unstructured activities (e.g. walking, cycling, swimming)
- For children under the age of 10, enjoyable and safe non-competitive activities should be preferred.

Examples of moderate - to vigorous-intensity physical activities:

- Cycling
- Playground activities
- Swimming
- Playing tag
- Brisk walking/jogging

Examples of vigorous-intensity activities, that strengthen muscle and bone:

- Climbing walls, trees, ropes
- Use of ladder
- Hopscotch
- Rope jumping
- Gymnastics, basketball, volleyball
- Yoga

Sleep habits

Children aged 9-12 years need 9-12 hours of sleep at a daily basis. To ensure optimum sleep quality:

- Turn off the TV and any other screen* at least 30 minutes before your child falls to sleep
- Remove the TV or any other screen from children's bedroom.
- Make sure the bedroom is as neat as possible .
- Maintain a steady sleep schedule and let your child become more responsible as he/she grows older.

**Screen is defined as: TV, PC, smartphones, tablets, videogames, etc.*

Sedentary activities

For children of this age it is recommended to:

- Reduce recreational screen* time to no more than 1-2 hours per day.
- Limit sedentary (motorized) transport.

How to limit sedentary time:

- Set specific hours throughout the day that children will spend on TV or other screen.
- Remove the TV or any other screen from children's bedroom.
- Instead of driving, walk to school or to extra curriculum activities.
- Take your pet for a walk along with your child.
- Get up along with your child and move indoors for 2-5 minutes after prolonged sitting time periods (duration ≥1 hour).
- Do not use screen time as punishment or reward (e.g. "You will watch TV if you do your homework".) .

**Screen is defined as: TV, PC, smartphones, tablets, videogames, etc.*

Dietary Recommendations for 9-12 years old children

Food Group	Recommended servings	Size of servings
Vegeta-bles	2-3 servings/ day	1 cup. cooked or raw vegetables,<br>2 cups raw green vegetables<br>1 medium cucumber,<br>1 big tomato or 1 cup grated tomato<br>2 medians carrots
Fruit	2-3 servings/ day	1 medium fruit (e.g. 1 apple),<br>2 small fruits (e.g. 2 tangerines),<br>30 small grapes,<br>1 slice of watermelon or melon,<br>4 dried fruits (e.g. plums),<br>1,5 tablespoon of raisins,<br>8 strawberries<br>15 cherries<br>½ cup (125 ml) fresh juice
Milk and dairy products	3-4 servings/ day	1 cup (250 ml) milk or ½ cup (125 mL) evaporated milk<br>1 cup (200 gr) yogurt<br>30gr hard cheese<br>2 tablespoons of soft cheese
Cereal (bread, rice, pas-ta) & po-tato	5-6 servings/ day	1 slice of bread (30 gr) or 2 small rusks or 1 medium rusk,<br>½ cup cereals<br>½ round sesame bread,<br>½ cup of cooked pasta or rice,
Legumes	At least 3 servings/ week	90-120g cooked and drained leg-umes
White and red meat	2-3 servings/ week	90-120g cooked meat
Fish and Seafood	2-3 servings/ week	120-150gr cooked fish without bones or cooked seafood
Eggs	4-7 / week	1 egg
Added fat and oils, olives and nuts	3-4 servings/ week	1 tablespoon of olive oil or other vegetable oil or butter or marga-rine, 1 handful of nuts (e.g. 18 al-monds, 6 whole walnuts, 3 table-spoons of sun flower seeds),<br>10-12 olives<br>1,5 tablespoon of tahini

Indicative Diet Meal Plan for children 9-12 years old

Breakfast.
- 1 cup milk (250ml) 1.5% fat or 1 cup yogurt 2% fat
- 1 slice of bread (30g) or 2 rusks or ½ cup of cereals

Morning Snack:
- 30g cheese (low fat) or cheese & turkey sliced or 1 egg
- 2 slices of bread (60g) or 4 rusks
- Vegetables (e.g. tomato, lettuce, cucumber)
- 1 fruit

Lunch:
- 80g red meat or chicken or fish
- Plenty of vegetables
- 3 teaspoons of olive oil
- 2/3 cup pasta or rice or 1 median potato or 1 cup legumes
- 1 slice of bread(30g) or 2 rusks
- 1 fruit

Afternoon Snack:
- 1 cup milk (250ml) 1.5% fat or 1 cup yogurt 2% fat
- 1 slice of bread (30g) or 2 rusks

Dinner:
- 55g red meat or chicken or fish
- Plenty of vegetables
- 3 teaspoons of olive oil
- ½ cup pasta or rice or 1 median potato or ¾ cup legumes
- 0,5 slice of bread (15g) or 1 rusk
- 1 fruit

Before Bedtime:
- 1 cup milk (250ml) 1.5% fat or 1 cup yogurt 2% fat

Source: Linou, A., et al. *National dietary guidelines for infants, children and adolescents.* National Die-tary Guidelines "Eu Dia...Trofin", pub. Institute of Preventive Medicine Environmental and Occupational Health Prolepsis. 2014: Labrakis Press S.A.

How can you increase fibers and complex carbohydrates in your child's diet and reduce the added sugars?

Carbohydrates are the main source of energy for your child's body. Therefore, it is recommended that 50% of the consumed carbohydrates should be of complex origin (i.e. not directly absorbed), as well as for your child to increase the consumption of fiber, due to their health benefits.

To increase the consumption of complex carbohydrates and fiber in your child's diet:

- Choose whole grains, such as bread, rusks, lebanese pita bread, pita bread, rice, pasta, vegetables and fruits, which are good sources of complex carbohydrates and fiber, in both main meals and snacks.
- If your child does not prefer whole grains, add them gradually to his diet. You can first mix processed cereals with whole grains and gradually decrease the proportion of processed cereals consumed in their meals.
- Select whole wheat flour when preparing meals (e.g. for homemade pies or pastries).
- Check the food labels and make sure that whole grains are at the top of the list of the product's ingredients . Make sure to also check its fiber content.
- Offer your child unpeeled fruits. Just make sure to give them a good solid rinse first.

To reduce the consumption of simple carbohydrates and added sugars from your child's diet:

- Discourage the frequent consumption of foods with added sugars, because children have an inherent preference for the sweet taste. More specifically, limit the consumption of foods such as:
  - ⇒ Sweets
  - ⇒ Cookies
  - ⇒ Yogurt desserts
  - ⇒ Packaged juices
  - ⇒ Soft Drinks
- Check the food labels and avoid buying foods when simple or added sugars are at the top on the ingredients' list.

How can you reduce bad fats in your child's diet

Saturated and trans fats belong in the bad fat category due to their harmful effects on health.

To reduce their intake from you child's diet:

- Offer to your child foods rich in good fats, such as fish, seafood and nuts.
- Use olive oil or soft vegetable fat spreads in your meals and avoid butter.
- Avoid consuming processed products such as fried foods and pastries that are rich in trans fats.
- Limit the consumption of foods rich in saturated fats, such as red meat, skin of meat and milk creams.
- Check the food labels and avoid foods that contain hydrogenated or trans fats. Also choose foods with low saturated fat concentration (<10%).

How can you reduce salt in your child's diet

Increased salt intake can elevate your child's blood pressure.

To reduce salt intake from you child's diet:

- Use herbs and spices instead of salt in your culinary preparations such as dill, oregano, basil, thyme, etc.
- Remove the salt from the table at meal time.
- Avoid consuming processed foods that are rich in salt, such as salty snacks, fast food, canned food and food preserved in salt.
- Be aware of the hidden salt sources, such as bread, pastries, sauces, mayonnaise, breakfast cereals, cereal bars, etc.
- Check the food labels of the products destined for your child and choose those that contain no salt or as little as possible.

Ten steps to Healthy Eating for children and adolescents

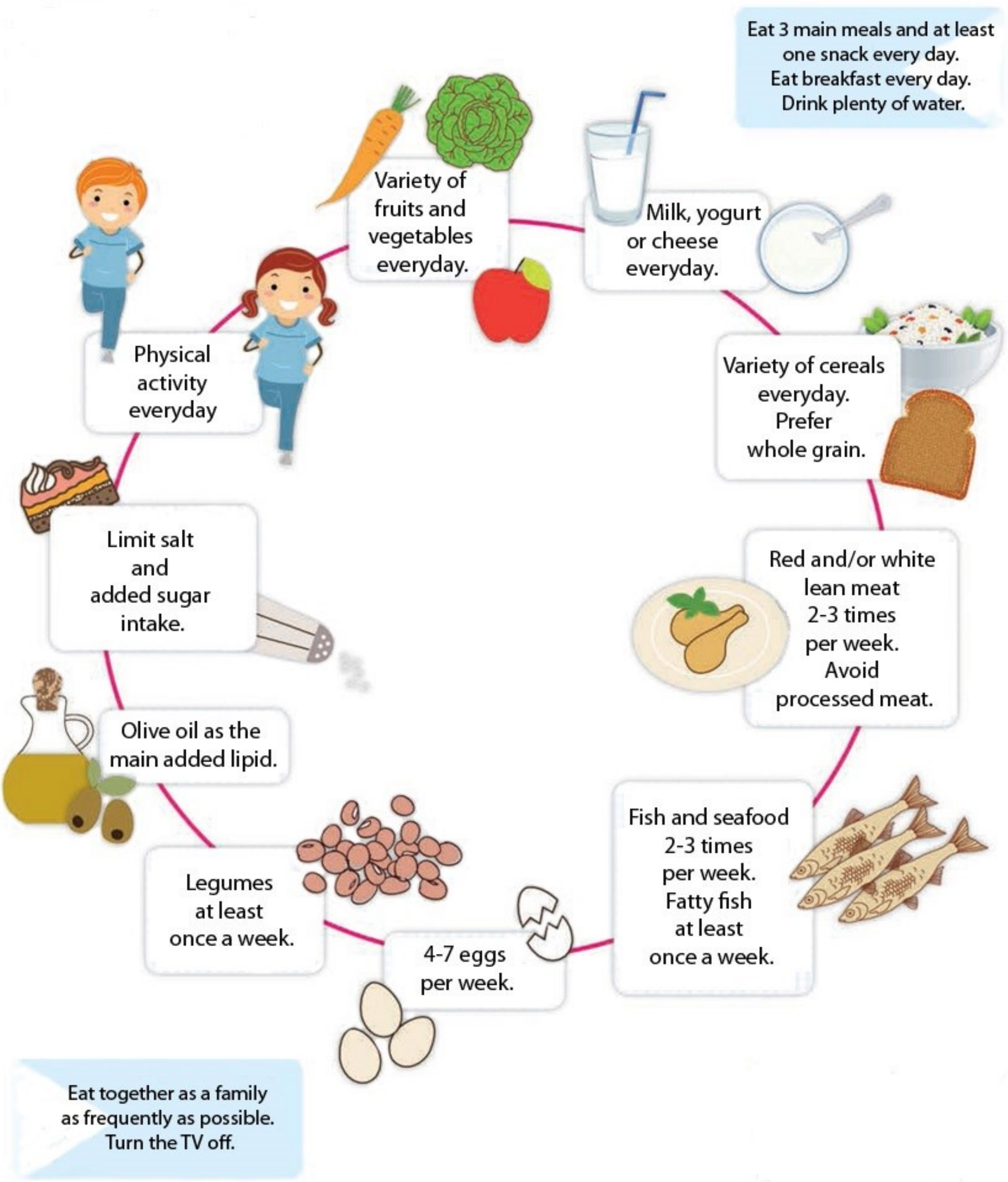

Source: Linou, A., et al. *National dietary guidelines for infants, children and adolescents*. National Dietary Guidelines "Eu Dia...Trofin", pub. Institute of Preventive Medicine Environmental and Occupational Health Prolepsis. 2014: Labrakis Press S.A.

children 9—12 years old

Tips towards a healthy lifestyle

The role of healthy nutrition

- Adopting a healthy nutrition is very important not only for the children's physical and mental health, as well as for the whole family's.
- Healthy dietary habits provide us with the energy and nutrients required daily at every age.
- Your child's nutrition at this stage contributes actively to his growth, since by the age of 10, a child's weight is increased by 3 kg per year and its height by about 6 cm, up to the pre-puberty period.
- Studies have shown that children consuming healthy foods have better memory, concentration, academic and athletic performance, as well as a more strengthened immune system than most children
- Dietary habits acquired during childhood are the cornerstone for maintaining health and well-being during adulthood.

Dietary tips for the whole family

- All family members should consume 3 main meals daily at regular intervals. Children should eat at least 1 small snack (e.g. 1 cup of yogurt with fruits), while adults can also eat snacks between main meals.
- Be a role model for your child by eating a balanced breakfast that includes foods from at least 3 food groups (dairy, fruit, cereal).
- Snacks may contain food choices from 2 or more food groups (e.g. round sesame bread and a fruit).
- Always have healthy homemade snacks (e.g. cut fruits, vegetables) at the ready to prevent them from choosing a less healthy snack.
- Limit the intake of fat, salt and added sugars.
- Make sure to eat as a whole family, without interference and turn the meals into a pleasant experience for everyone .
- Encourage children to try new foods by repeated exposure .
- Include children at all stages of a meal preparation from an early age.
- Serve the same meal to all family members. Avoid preparing something special for children.
- Limit take-out to a minimum of 1 per week
- Do not use sweets as rewards.

Physical activity guidelines for children

Children at this age are recommended to:

- Accumulate at least ***60 minutes*** of moderate - to vigorous-intensity physical activity ***daily*** either continuously or in multiple shorter bouts of at least 10 minutes spread throughout the day.
- Participate in vigorous-intensity activities, including those that strengthen muscle and bone, at ***least 3 times per week***.
- Physical activities include either structured (e.g. sports) or unstructured activities (e.g. walking, cycling, swimming)
- For children under the age of 10, enjoyable and safe non-competitive activities should be preferred.

Examples of moderate - to vigorous-intensity physical activities:

- Cycling
- Playground activities
- Swimming
- Playing tag
- Brisk walking/jogging
- Ice-skating or road-skating
- Dancing

Examples of vigorous-intensity activities, that strengthen muscle and bone:

- Climbing walls, trees, ropes
- Use of ladder
- Hopscotch
- Rope jumping
- Gymnastics, basketball, volleyball
- Yoga
- Martial arts
- Running

Examples of moderate intensity physical activities for adults:

- Brisk walking
- Dancing
- Gardening
- Household activities
- Active play with children
- Walk with pet
- Cycling <15km/hour
- Carrying load <20kg

Examples of vigorous intensity physical activities for adults:

- Running
- Walking on a slope
- Cycling >15km/hour
- Swimming
- Aerobics
- Competitive sports (basketball, football, etc.)
- Carrying load >20kg

Physical activity guidelines for adults

For adults aged 18-64 years it is recommended to:

- Do **at least 150 minutes** of moderate-intensity aerobic physical activity **throughout the week (or 30 minutes per day)** or do **at least 75 minutes** of vigorous-intensity aerobic physical activity **throughout the week (or 15 minutes per day** either continuously or in multiple shorter bouts of at least 10 minutes spread throughout the day.
- Participate in muscle-strengthening activities **on 2 or more days a week.**

Sedentary activities for children

For children of this age it is recommended to:

- Reduce recreational screen* time to **no more than 1-2 hours per day**.
- Limit sedentary (motorized) transport.

Sleep habits for children

Children aged 9-12 years need 9-12 hours of sleep at a daily basis. To ensure optimum sleep quality:

- Turn off the TV and any other screen* at least 30 minutes before your child falls to sleep
- Remove the TV or any other screen from children's bedroom.
- Make sure the bedroom is as neat as possible .
- Maintain a steady sleep schedule and let your child become more responsible as he/she grows older.

** Screen is defined as: TV, PC, smartphones, tablets, video-games, etc..*

Sedentary activities for adults

For adults it is recommended:

- Limit sedentary habits as much as possible and make regular movement breaks.

How to limit sedentary time:

- Set specific hours throughout the day that family members will spend on TV or other screen
- Remove the TV or any other screen from family members' bedroom
- Instead of driving, use public transportation and get off the bus/metro 2-3 stops before your final destination.
- Instead of driving, walk to school or to extra curriculum activities along with your child.
- Take your pet for a walk along with your child.
- Get up along with your child and move indoors for 2-5 minutes after prolonged sitting time periods (duration ≥1 hour).
- Do not use screen time as punishment or reward (e.g. "You will watch TV if you do your homework".).

Dietary Recommendations for 9-12 years old children

Food Group	Recommended servings	Size of servings
Vegetables	2-3 servings/ day	1 cup. cooked or raw vegetables,<br>2 cups raw green vegetables<br>1 medium cucumber,<br>1 big tomato or 1 cup grated tomato<br>2 medians carrots
Fruit	2-3 servings/ day	1 medium fruit (e.g. 1 apple),<br>2 small fruits (e.g. 2 tangerines),<br>30 small grapes,<br>1 slice of watermelon or melon,<br>4 dried fruits (e.g. plums),<br>1,5 tablespoon of raisins,<br>8 strawberries<br>15 cherries<br>½ cup (125 ml) fresh juice
Milk and dairy products	3-4 servings/ day	1 cup (250 ml) milk or ½ cup (125 mL) evaporated milk<br>1 cup (200 gr) yogurt<br>30gr hard cheese<br>2 tablespoons of soft cheese
Cereal (bread, rice, pasta) & potato	5-6 servings/ day	1 slice of bread (30 gr) or 2 small rusks or 1 medium rusk,<br>½ cup cereals<br>½ round sesame bread,<br>½ cup of cooked pasta or rice,<br>1 medium cooked potato
Legumes	At least 3 servings/ week	90-120g cooked and drained legumes
White and red meat	2-3 servings/ week	90-120g cooked meat
Fish and Seafood	2-3 servings/ week	120-150gr cooked fish without bones or cooked seafood
Eggs	4-7 / week	1 egg
Added fat and oils, olives and nuts	3-4 servings/ week	1 tablespoon of olive oil or other vegetable oil or butter or margarine, 1 handful of nuts (e.g. 18 almonds, 6 whole walnuts, 3 tablespoons of sun flower seeds),<br>10-12 olives<br>1,5 tablespoon of tahini

Indicative Diet Meal Plan for children 9-12 years old

Breakfast.

- 1 cup milk (250ml) 1.5% fat or 1 cup yogurt 2% fat
- 1 slice of bread (30g) or 2 rusks or ½ cup of cereals

Morning Snack:

- 30g cheese (low fat) or cheese & turkey sliced or 1 egg
- 2 slices of bread (60g) or 4 rusks
- Vegetables (e.g. tomato, lettuce, cucumber)
- 1 fruit

Lunch:

- 80g red meat or chicken or fish
- Plenty of vegetables
- 3 teaspoons of olive oil
- 2/3 cup pasta or rice or 1 median potato or 1 cup legumes
- 1 slice of bread(30g) or 2 rusks
- 1 fruit

Afternoon Snack:

- 1 cup milk (250ml) 1.5% fat or 1 cup yogurt 2% fat
- 1 slice of bread (30g) or 2 rusks

Dinner:

- 55g red meat or chicken or fish
- Plenty of vegetables
- 3 teaspoons of olive oil
- ½ cup pasta or rice or 1 median potato or ¾ cup legumes
- 0,5 slice of bread (15g) or 1 rusk
- 1 fruit

Before Bedtime:

- 1 cup milk (250ml) 1.5% fat or 1 cup yogurt 2% fat

Source: Linou, A., et al. *National dietary guidelines for infants, children and adolescents*. National Dietary Guidelines "Eu Dia...Trofin", pub. Institute of Preventive Medicine Environmental and Occupational Health Prolepsis. 2014: Labrakis Press S.A.

Dietary Recommendations for Adults

Food Group	Recommended servings	Size of servings
Vegetables	4 servings/ day	1 cup. cooked or raw vegetables,<br>2 cups raw green vegetables<br>1 medium cucumber,<br>1 big tomato or 1 cup grated tomato<br>2 medians carrots
Fruit	3 servings/ day	1 medium fruit (e.g. 1 apple),<br>2 small fruits (e.g. 2 tangerines),<br>30 small grapes,<br>1 slice of watermelon or melon,<br>4 dried fruits (e.g. plums),<br>1,5 tablespoon of raisins,<br>8 strawberries<br>15 cherries<br>½ cup (125 ml) fresh juice
Milk and dairy products	2 servings/ day	1 cup (250 ml) milk or ½ cup (125 mL) evaporated milk<br>1 cup (200 gr) yogurt<br>30gr hard cheese<br>2 tablespoons of soft cheese
Cereal (bread, rice, pasta) & potato	5-8 servings / day of which potato consumption is about 3 servings per week	1 slice of bread (30 gr) or 2 small rusks or 1 medium rusk,<br>½ cup cereals<br>½ round sesame bread,<br>½ cup of cooked pasta or rice,<br>1 medium cooked potato
Legumes	At least 3 servings/ week	150–200g cooked and drained legumes (1 cup)
Red meat	1 servings/ week	120-150g red cooked meat
White meat	1-2 servings/ week	120-150g white cooked meat
Fish and Seafood	2-3 servings /week, at least half of the servings are oily fish (eg, sardine, anchovy, coli, zaranana), which are rich in omega-3 fat.	150g of cooked fish or seafood (eg 1 medium sea bream, 10-12 anchovies, 10-12 sardines, 15 medium shrimps, 12-14 big mussels)
Eggs	3-4 servings/ week	1 egg
Added fat and oils, olives and nuts	4-5 servings/ day (consume olive oil as the first choice of added oil in cooking and salad)	1 tablespoon of olive oil or other vegetable oil or butter or margarine, 1 handful of nuts (e.g. 18 almonds, 6 whole walnuts, 3 tablespoons of sun flower seeds),<br>10-12 olives<br>1,5 tablespoon of tahini

Source: Linou, A., et al. *National dietary guidelines for infants, children and adolescents*. National Dietary Guidelines "Eu Dia...Trofin", pub. Institute of Preventive Medicine Environmental and Occupational Health Prolepsis. 2014: Labrakis Press S.A.

Indicative Diet Meal Plan for Adults

Breakfast:
- 1 cup milk (240ml) 1.5% fat or 1 cup yogurt 2% fat
- 1 cup of cereals or 4 rusks or 2 slices of bread

Lunch:
- 150g fish or chicken or turkey or beef or pork
- 1 cup of rice or pasta or 2 small potatoes or 2 slices of bread
- 2 cups of raw vegetables or 1 cup of boiled vegetables
- 1 tablespoon of olive oil
- 1 slice of bread
- 1 fruit

Or

- 1 cup legumes (e.g. lentils, chickpeas, bean soup, giants beans, split peas) with 1 tablespoon of olive oil / serving
- 60g cheese or 30g cheese with 1 egg
- 5 big or 10 small olives
- 1 slice of bread
- 1 fruit

Or

- 1 cup of cooked vegetables (π.χ. okra, artichokes, eggplant, green beans) with ½ cup of potatoes (or 1 small potato) with 1 tablespoon of olive oil
- 90g fish or 90g chicken or 60g cheese and 1 egg
- 2 slices of bread
- 1 fruit

Dinner:
- 120g chicken or 120g tuna or 120g cheese or 2 eggs
- 1 cup of rice or pasta or 2 small potatoes or 2 slices of bread
- 2 cups of raw vegetables or 1 cup of boiled vegetables
- 1 tablespoon of olive oil
- 1 fruit

Or

- 2 slices of bread, 2 slices of cheese (60g) and 2 slices of turkey (60g)
- 2 cups of raw vegetables or 1 cup of boiled vegetables
- 1 tablespoon of olive oil
- 1 fruit

Snack (Consumed during the day) :
- 1 fruit
- 4 crackers ή 1 sesame round bread
- 1 cup milk (240ml) 1.5% fat or 1 cup yogurt 2% fat

How can you increase fibers and complex carbohydrates in your family's diet and reduce the added sugars?

Carbohydrates are the main source of energy for your body. Therefore, it is recommended that 50% of the consumed carbohydrates should be of complex origin (i.e. not directly absorbed), as well as to increase the consumption of fiber, due to their health benefits.

To increase the consumption of complex carbohydrates and fiber in your family's diet:

- Choose whole grains, such as bread, rusks, lebanese pita bread, pita bread, rice, pasta, vegetables and fruits, which are good sources of complex carbohydrates and fiber, in both main meals and snacks.
- If your family members do not prefer whole grains, add them gradually to their diet. You can first mix processed cereals with whole grains and gradually decrease the proportion of processed cereals consumed in their meals.
- Select whole wheat flour when preparing meals (e.g. for homemade pies or pastries).
- Check the food labels and make sure that whole grains are at the top of the list of the product's ingredients . Make sure to also check its fiber content.
- Eat unpeeled fruits. Just make sure to give them a good solid rinse first.

Limit the consumption of simple carbohydrates and added sugars from your family's diet, specially for children that have an inherent preference for the sweet taste.

- More specifically, limit the consumption of foods such as:
  - ⇒ Sweets
  - ⇒ Cookies
  - ⇒ Yogurt desserts
  - ⇒ Packaged juices
  - ⇒ Soft drinks
- Check the food labels and avoid buying foods when simple or added sugars are at the top on the ingredients' list.

How can you reduce bad fats in your family's diet

Saturated and trans fats belong in the bad fat category due to their harmful effects on health.

To reduce their intake from you family's diet:

- Eat foods rich in good fats, such as fish, seafood and nuts.
- Use olive oil or soft vegetable fat spreads in your meals and avoid butter.
- Avoid consuming processed products such as fried foods and pastries that are rich in trans fats.
- Limit the consumption of foods rich in saturated fats, such as red meat, skin of meat and milk creams.
- Check the food labels and avoid foods that contain hydrogenated or trans fats. Also choose foods with low saturated fat concentration (<10%).

How can you reduce salt in your family's diet

Increased salt intake can elevate your blood pressure at any age.

To reduce salt intake from you family's diet:

- Use herbs and spices instead of salt in your culinary preparations such as dill, oregano, basil, thyme, etc.
- Remove the salt from the table at meal time.
- Avoid consuming processed foods that are rich in salt, such as salty snacks, fast food, canned food and food preserved in salt.
- Be aware of the hidden salt sources, such as bread, pastries, sauces, mayonnaise, breakfast cereals, cereal bars, etc.
- Check the food labels of the products you buy and choose those that contain no salt or as little as possible.

Ten steps to Healthy Eating for the whole family

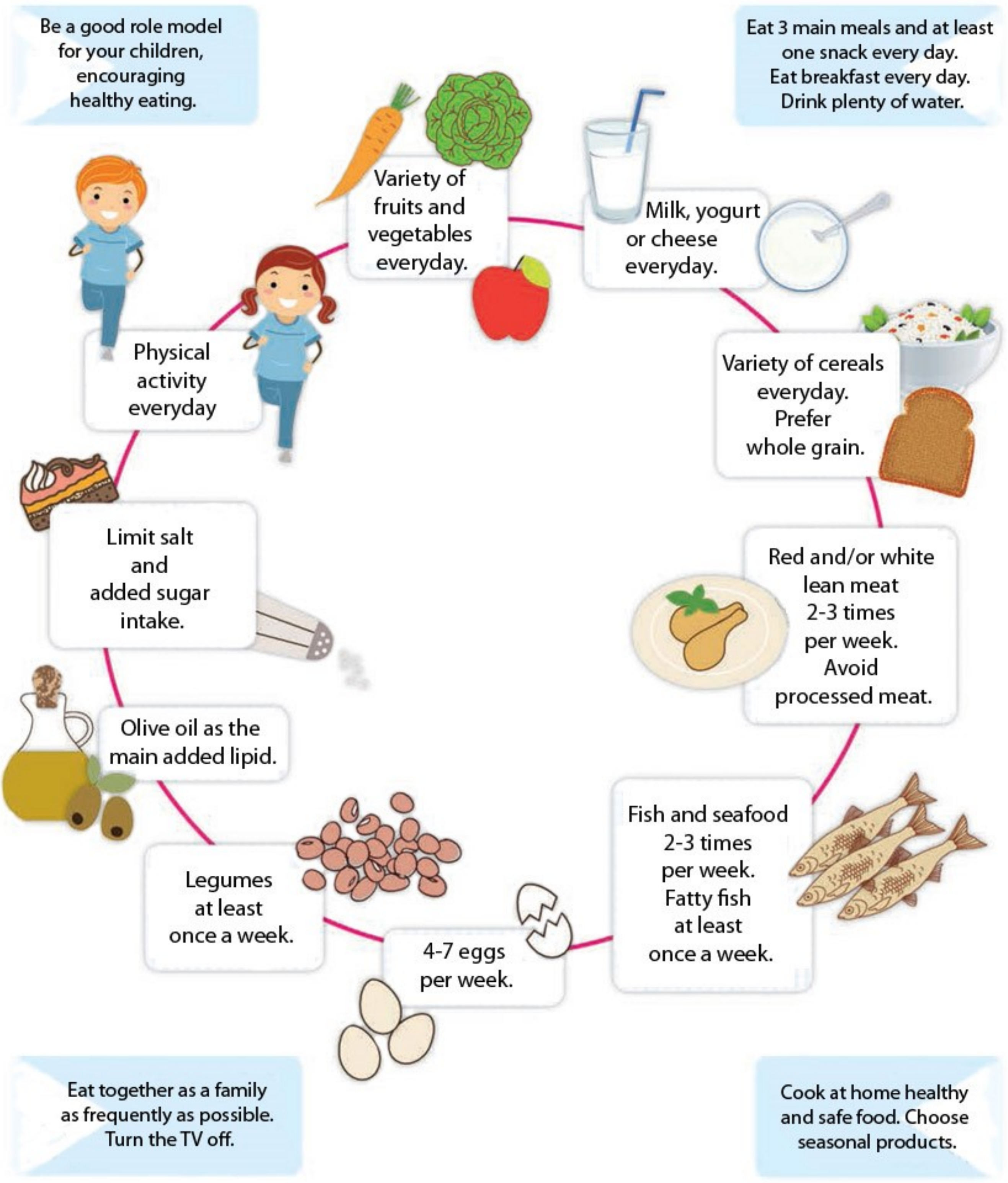

Source: Linou, A., et al. *National dietary guidelines for infants, children and adolescents*. National Dietary Guidelines "Eu Dia...Trofin", pub. Institute of Preventive Medicine Environmental and Occupational Health Prolepsis. 2014: Labrakis Press S.A.
