# Supplementary material for: Assessment of the Effectiveness of a Computerised Decision-Support Tool for Health Professionals for the Prevention and Treatment of Childhood Obesity. Results from a Randomised Controlled Trial
Source: Nutrients. 2019 Mar 26;11(3):706. doi: 10.3390/nu11030706 (PMC6471646; doi:10.3390/nu11030706)
Supplement: Supplementary file 1 [file nutrients-11-00706-s001.zip › nutrients-456694-SI/Supplementary Material/Supplementary Table S2 Example meal plans 1600 kcals.pdf]

Diet Meal Plan 1

**Day 1st**	**Day 2nd**	**Day 3rd**	**Day 4th**
Breakfast<br>• 1 cup of milk (250ml) 1.5% fat or 1 cup (200g) of yogurt 2% fat<br>• ½ cupofcereals (preferably whole wheat)	Breakfast<br>• 1 cupof milk (250ml) 1.5% fat or 1 cup (200g) of yogurt 2% fat<br>• 2 rusks (preferably whole wheat) with 2 teaspoons of jam	Breakfast<br>• 1 cup of milk (250ml) 1.5% fat or 1 cup (200g) of yogurt 2% fat<br>• 2 rusks (preferably whole wheat) with 2 teaspoons of tahini	Breakfast<br>• 1 cup of milk (250ml) 1.5% fat or 1 cup (200g) of yogurt 2% fat<br>• ½ cup of cereals (preferably whole wheat)
Morning Snack(school)<br>• Sandwich with 2 slices of bread (preferably whole wheat) and 1 slice of cheese (low fat) and vegetables (e.g. tomato, lettuce, cucumber) (1stbreak)<br>• 1 medium sized fruit (2nd or 3rd break)	Morning Snack (school)<br>• Sandwich with 2 slices of bread (preferably whole wheat) and 1 slice of cheese (low fat) and vegetables (e.g. tomato, lettuce, cucumber) (1st break)<br>• 1 medium sized fruit (2nd or 3rd break)	Morning Snack (school)<br>• 1 round sesame bread (60g) (1st break)<br>• 1 medium sized fruit (2nd or 3rd break)	Morning Snack(school)<br>• 1 round sesame bread (60g) (1st break)<br>• 1 medium sized fruit (2nd or 3rd break)
Lunch<br>• 90g chicken's leg without skin cooked with lemon or tomato (homemade tomato sauce) with 1 teaspoon of olive oil<br>• 1 medium sized baked potato with 1 teaspoon of olive oil<br>• Fresh salad made with raw or boiled vegetables with 1 teaspoon of olive oil	Lunch<br>• 1 cup of pasta cooked with 1 teaspoon of olive oil<br>• 30g gratedcheese (preferably low fat)<br>• Fresh salad made with raw or boiled vegetables with 2 teaspoons of olive oil	Lunch<br>Omelet (ingredients):<br>• 2eggs<br>• Vegetables (e.g. mushrooms, peppers, tomato)<br>• 1 teaspoon of olive oil<br>• 1 slice of bread (30g) (preferably whole wheat)<br>• Fresh salad made with raw or boiled vegetables with 2 teaspoons of olive oil<br>• 1 medium sized fruit	Lunch<br>• 1 cup of bean soup cooked with 2 teaspoons of olive oil<br>• 30g cheese (low fat)<br>• Fresh salad made with raw or boiled vegetables with 1 teaspoon of olive oil
Afternoon Snack<br>• 1 cup of milk (250ml) 1.5% fat or 1cup (200g) of yogurt 2% fat<br>• 1 medium sized fruit	Afternoon Snack<br>• 1 cup of milk (250ml) 1.5% fat or 1 cup (200g) of yogurt 2% fat<br>• 1 medium sized fruit	Afternoon Snack<br>• 1 cup of milk (250ml) 1.5% fat or 1 cup (200g) of yogurt 2% fat<br>• ½ cup of cereals (preferably whole wheat)	Afternoon Snack<br>• 1 cup of milk (250ml) 1.5% fat or 1 cup (200g) of yogurt 2% fat<br>• 1 medium sized fruit
Dinner<br>Greek Dakos salad (ingredients):<br>• 1 medium sized rusk (60g)<br>• 30g cheese (low fat)<br>• grated or chopped tomato<br>• 2 teaspoons of olive oil<br>• 1 medium sized fruit	Dinner<br>• Caesar's Salad (ingredients):<br>• plenty of vegetables<br>• 30g grated cheese (preferably low fat)<br>• 30g chicken chopped<br>• ½cup of croutons<br>• 2 teaspoons of olive oil or 4 teaspoons of mayonnaise<br>• 1 medium sized fruit	Dinner<br>• Sandwich with 2 slices of bread (preferably whole wheat) and 1 slice of cheese (low fat) and 1 slice of turkey boiled and vegetables (e.g. Tomato, lettuce, cucumber)<br>• 1 medium sized fruit	Dinner<br>Chef's salad:<br>• 1 egg boiled<br>• 30g grated cheese (low fat)<br>• 30g turkey boiled chopped<br>• plenty of vegetables<br>• ½ cup of croutons<br>• 2 teaspoons of olive oil or 4 teaspoons of mayonnaise
Before Bedtime<br>• 1 cup of milk (250ml) 1.5% fat or 1 cup (200g) of yogurt 2% fat	Before Bedtime<br>• 1 cup of milk (250ml) 1.5% fat or 1 cup (200g) of yogurt 2% fat	Before Bedtime<br>• 1 cup of milk (250ml) 1.5% fat or 1 cup (200g) of yogurt 2% fat	Before Bedtime<br>• 1 cup of milk (250ml) 1.5% fat or 1 cup (200g) of yogurt 2% fat

Diet Meal Plan 1

Day 5th	Day 6th Weekend	Day 7th Weekend
Breakfast<br>• 1 cup of milk (250ml) 1.5% fat or 1 cup (200g) of yogurt 2% fat<br>• ½ cup of cereals (preferably whole wheat)	Breakfast<br>• 1 cupof milk (250ml) 1.5% fat or 1 cup (200g) of yogurt 2% fat<br>• ½ cupofcereals (preferably whole wheat)	Breakfast<br>• 1 cupof milk (250ml) 1.5% fat or 1 cup (200g) of yogurt 2% fat<br>• ½cupofcereals (preferably whole wheat)
Morning Snack(school)<br>• Sandwich with 2 slices of bread (preferably whole wheat) and 1 slice of cheese (low fat) and vegetables (e.g. tomato, lettuce, cucumber) (1stbreak)<br>• 1 medium sized fruit (2nd or 3rd break)	Morning Snack<br>• Sandwich with 2 slices of bread (preferably whole wheat) and 4 teaspoons of tahini<br>• 1 medium sized fruit	Morning Snack<br>• Sandwich with 2 slices of bread (preferably whole wheat) and 4 teaspoons of tahini<br>• 1 medium sized fruit
Lunch<br>• 1 cup of green beans cooked with 2 teaspoons of olive oil<br>• 1 medium sized baked potato<br>• Fresh salad made with raw or boiled vegetables with 1 teaspoon of olive oil<br>• 1 slice of bread (30g) (preferably whole wheat)	Lunch<br>• 90g fish roasted with 1 teaspoon of olive oil<br>• ½cup of rice boiled<br>• Fresh saladmade withraw or boiled vegetables with 2 teaspoons of olive oil	Lunch<br>• 90g shrimps cooked with homemade tomato sauce and 1 teaspoon of olive oil<br>• 1 cup of pasta cooked (preferably whole wheat)<br>• 1 tablespoon of grated cheese<br>• Fresh saladmade withraw or boiled vegetables with 1 teaspoon of olive oil
Afternoon Snack<br>• 1 cup of milk (250ml) 1.5% fat or 1 cup (200g) of yogurt 2% fat<br>• 1 medium sized fruit	Afternoon Snack<br>• 1 cupof milk (250ml) 1.5% fat or 1 cup (200g) of yogurt 2% fat<br>• 1 medium sized fruit	Afternoon Snack<br>• 1 cupof milk (250ml) 1.5% fat or 1cup (200g) of yogurt 2% fat<br>• 1 medium sized fruit
Dinner<br>• 1 chicken souvlaki roasted (100g)<br>• Fresh salad made with raw or boiled vegetables with 2 teaspoons of olive oil<br>• 1pita bread (60g) without olive oil	Dinner<br>Homemade Pizza (ingredients):<br>• 1 pita bread (60g)<br>• vegetables (e.g. tomato, peppers, onions, mushrooms)<br>• 30g grated cheese (low fat)<br>• 1 slice of turkey boiled chopped	Dinner<br>• Sandwichwith 2 slicesofbread (preferably whole wheat),1 slice of cheese (low fat) and 1 slice of turkey boiled and vegetables (e.g. tomato, lettuce, cucumber)
Before Bedtime<br>• 1 cup of milk (250ml) 1.5% fat or 1 cup (200g) of yogurt 2% fat	Before Bedtime<br>• 1 cupof milk (250ml) 1.5% fat or 1 cup (200g) of yogurt 2% fat	Before Bedtime<br>• 1 cupof milk (250ml) 1.5% fat or 1 cup (200g) of yogurt 2% fat

Comments

(In the box below please record if you do anything different from what is recommended in your diet meal plan)

Diet Meal Plan 2

**Day 1st**	**Day 2nd**	**Day 3rd**	**Day 4th**
Breakfast<br>• 1 cup of milk (250ml) 1.5% fat or 1 cup (200g) of yogurt 2% fat<br>• ½ cup of cereals (whole wheat)	Breakfast<br>• 1 cup of milk (250ml) 1.5% fat or 1 cup (200g) of yogurt 2% fat<br>• 2 rusks (whole wheat) with 1 teaspoon of jam	Breakfast<br>• 1 cup of milk (250ml) 1.5% fat or 1 cup (200g) of yogurt 2% fat<br>• 2 rusks (whole wheat) with 2 teaspoons tahini	Breakfast<br>• 1 cup of milk (250ml) 1.5% fat or 1 cup (200g) of yogurt 2% fat<br>• ½ cup of cereals (whole wheat)
Morning Snack (school)<br>• Sandwich with 2 slices of bread (whole wheat) and 1 slice of cheese (low fat) and vegetables (e.g. tomato, lettuce, cucumber) (1st break)<br>• 1 medium sized fruit (2nd or 3rd break)	Morning Snack (school)<br>• Sandwich with 2 slices of bread (toasted) (whole wheat) and 1 slice of cheese (low fat) and vegetables (e.g. tomato, lettuce, cucumber) (1st break)<br>• 1 medium sized fruit (2nd or 3rd break)	Morning Snack (school)<br>• 1 round sesame bread (60g) (1st break)<br>• 1 medium sized fruit (2nd or 3rd break)	Morning Snack (school)<br>• 1 round sesame bread (60g) (1st break)<br>• 1 medium sized fruit (2nd or 3rd break)
Lunch<br>• 90g salmon roasted<br>• 1/3 cup rice boiled (preferably brown) cooked with 1 teaspoon of olive oil<br>• Fresh salad made with raw or boiled vegetables with 2 teaspoons olive oil	Lunch<br>• 1 cup pasta cooked with 1 teaspoon of olive oil<br>• 60g grated cheese (preferably low fat)<br>• Fresh salad made with raw or boiled vegetables with 1 teaspoon olive oil	Lunch<br>Omelet (ingredients):<br>• 2 eggs white and 1 egg yolk<br>• vegetables (e.g. mushrooms, peppers, tomato)<br>• 30g grated cheese (low fat)<br>• 2 teaspoons of olive oil<br>• 1 slice of bread (30g) (whole wheat)<br>• Fresh salad made with raw or boiled vegetables with 1 teaspoon olive oil	Lunch<br>• 1 cup of chickpeas cooked with 2 teaspoons of olive oil<br>• 30g cheese (low fat)<br>• Fresh salad made with raw or boiled vegetables with 1 teaspoon olive oil<br>• 1 medium sized fruit
Afternoon Snack<br>• 1 cup of milk (250ml) 1.5% fat or 1 cup (200g) of yogurt 2% fat<br>• 1 medium sized fruit	Afternoon Snack<br>• 1 cup of milk (250ml) 1.5% fat or 1 cup (200g) of yogurt 2% fat<br>• 1 medium sized fruit	Afternoon Snack<br>• 1 cup of milk (250ml) 1.5% fat or 1 cup (200g) of yogurt 2% fat<br>• ½ cup of cereals (whole wheat)	Afternoon Snack<br>• 1 cup of milk (250ml) 1.5% fat or 1 cup (200g) of yogurt 2% fat<br>• 1 medium sized fruit
Dinner<br>Greek Dakos Salad (ingredients):<br>• 1 medium sized rusk (60g)<br>• 30g cheese (low fat)<br>• grated or chopped tomato<br>• 2 teaspoons olive oil<br>• 1 medium sized fruit	Dinner<br>• Caesar's Salad (ingredients):<br>• lettuce<br>• 30g grated cheese (low fat)<br>• 1 egg boiled<br>• 30g turkey boiled and chopped<br>• ½ cup. croutons whole wheat<br>• 2 teaspoons of olive oil or 2 teaspoons of mayonnaise light	Dinner<br>• Sandwich with 2 slices of bread (whole wheat) and 1 slice of cheese (low fat), 1 slice of turkey boiled and vegetables (e.g. tomato, lettuce, cucumber)<br>• 1 medium sized fruit	Dinner<br>Chef's salad:<br>• 1 egg boiled<br>• 30g grated cheese (low fat)<br>• 30g turkey boiled chopped<br>• plenty of vegetables<br>• ½ cup of croutons<br>• 2 teaspoons of olive oil or 4 teaspoons of mayonnaise
Before Bedtime<br>• 1 cup of milk (250ml) 1.5% fat or 1 cup (200g) of yogurt 2% fat	Before Bedtime<br>• 1 cup of milk (250ml) 1.5% fat or 1 cup (200g) of yogurt 2% fat	Before Bedtime<br>• 1 cup of milk (250ml) 1.5% fat or 1 cup (200g) of yogurt 2% fat	Before Bedtime<br>• 1 cup of milk (250ml) 1.5% fat or 1 cup (200g) of yogurt 2% fat

Diet Meal Plan 2

**Day 5th**	**Day 6th Weekend**	**Day 7th Weekend**
Breakfast<br>• 1 cup of milk (250ml) 1.5% fat or 1 cup (200g) of yogurt 2% fat<br>• ½ cup of cereals (whole wheat)	Breakfast<br>• 1 cup of milk (250ml) 1.5% fat or 1 cup (200g) of yogurt 2% fat<br>• ½ cup of cereals (whole wheat)	Breakfast<br>• 1 cup of milk (250ml) 1.5% fat or 1 cup (200g) of yogurt 2% fat<br>• ½ cup of cereals (whole wheat)
Morning Snack (school)<br>• Sandwich with 2 slices of bread (whole wheat) and 1 slice of cheese (low fat) and vegetables (e.g. tomato, lettuce, cucumber) (1st break)<br>• 1 medium sized fruit (2nd or 3rd break)	Morning Snack<br>• Sandwich with 2 slices of bread (whole wheat) and 4 teaspoons of tahini and 2 teaspoons honey<br>• 1 medium sized fruit	Morning Snack<br>• Sandwich with 2 slices of bread (preferably whole wheat) and 4 teaspoons of tahini and 2 teaspoons honey<br>• 1 medium sized fruit
Lunch<br>• 1 cup peas cooked with fresh tomato sauce and 2 teaspoons of olive oil<br>• Fresh salad made with raw or boiled vegetables with 1 teaspoon olive oil<br>• 1 slice of bread (30g) (whole wheat)<br>• 1 medium sized fruit	Lunch<br>• 90g sardine roasted<br>• ½ cup of rice boiled (preferably brown)<br>• Fresh salad made with raw or boiled vegetables with 1 teaspoon olive oil	Lunch<br>• 90g chicken's leg cooked with homemade tomato sauce or lemon and 1 teaspoon of olive oil<br>• 1 medium sized baked potato<br>• Fresh salad made with raw or boiled vegetables with 1 teaspoon olive oil
Afternoon Snack<br>• 1 cup of milk (250ml) 1.5% fat or 1 cup (200g) of yogurt 2% fat<br>• 1 medium sized fruit	Afternoon Snack<br>• 1 cup of milk (250ml) 1.5% fat or 1 cup (200g) of yogurt 2% fat<br>• 1 medium sized fruit	Afternoon Snack<br>• 1 cup of milk (250ml) 1.5% fat or 1 cup (200g) of yogurt 2% fat<br>• 1 medium sized fruit
Dinner<br>• 1 chicken souvlaki roasted (110g)<br>• Fresh salad made with raw or boiled vegetables with 1 teaspoon olive oil<br>• ½ pitta bread whole wheat (30g) without olive oil	Dinner<br>Homemade Pizza (ingredients):<br>• 1 pita bread whole bread(60g)<br>• vegetables (e.g. tomato, peppers, onions, mushrooms)<br>• 30g grated cheese (low fat)<br>• 1 slice of turkey boiled chopped	Dinner<br>Homemade Pizza (ingredients):<br>• 1 pita bread whole bread(60g)<br>• vegetables (e.g. tomato, peppers, onions, mushrooms)<br>• 30g grated cheese (low fat)<br>• 1 slice of turkey boiled chopped
Before Bedtime<br>• 1 cup of milk (250ml) 1.5% fat or 1 cup (200g) of yogurt 2% fat	Before Bedtime<br>• 1 cup of milk (250ml) 1.5% fat or 1 cup (200g) of yogurt 2% fat	Before Bedtime<br>• 1 cup of milk (250ml) 1.5% fat or 1 cup (200g) of yogurt 2% fat

Comments

(In the box below please record if you do anything different from what is recommended in your diet meal plan)

Diet Meal Plan 3

Day 1st

Breakfast
- 1 cup of milk (250ml) 1.5% fat or 1 cup (200g) of yogurt 2% fat
- ½ cup of cereals (whole wheat)

Morning Snack (school)
- Sandwich with 2 slices of bread (whole wheat), 30g cottage cheese and vegetables (e.g. tomato, lettuce, cucumber) (1st break)
- 1 medium sized fruit (2nd or 3rd break)

Lunch
- 90g roasted chicken's breast cooked with tomato sauce and herbs
- 3/4 cup rice boiled cooked with 1 teaspoon of olive oil
- Fresh salad made with raw or boiled vegetables with 2 teaspoons of olive oil

Afternoon Snack
- 1 cup of milk (250ml) 1.5% fat or 1 cup (200g) of yogurt 2% fat
- 1 medium sized fruit

Dinner

Greek Dakos salad (ingredients):
- 1 medium sized rusk whole wheat (60g)
- 30g cottage cheese
- grated or chopped tomato
- 2 teaspoons of olive oil
- 1 medium sized fruit

Before Bedtime
- 1 cup of milk (250ml) 1.5% fat or 1 cup (200g) of yogurt 2% fat

Day 2nd

Breakfast
- 1 cup of milk (250ml) 1.5% fat or 1 cup (200g) of yogurt 2% fat
- ½ cup of cereals (whole wheat)

Morning Snack (school)
- Sandwich with 2 slices of bread (whole wheat), 30g cottage cheese and vegetables (e.g. tomato, lettuce, cucumber) (1st break)
- 1 medium sized fruit (2nd or 3rd break)

Lunch
- 1 cup pasta (whole wheat) cooked with 1 teaspoon of olive oil
- 30g grated cheese (low fat)
- Fresh salad made with raw or boiled vegetables with 2 teaspoons of olive oil

Afternoon Snack
- 1 cup of milk (250ml) 1.5% fat or 1 cup (200g) of yogurt 2% fat
- 1 medium sized fruit

Dinner
- Caesar's Salad (ingredients):
  - lettuce
  - 30g cottage cheese
  - 30g roasted chicken's breast chopped
  - 2 grated rusks
  - 2 teaspoons of olive oil

Before Bedtime
- 1 cup of milk (250ml) 1.5% fat or 1 cup (200g) of yogurt 2% fat

Day 3rd

Breakfast
- 1 cup of milk (250ml) 1.5% fat or 1 cup (200g) of yogurt 2% fat
- 2 rusks (whole wheat) with 2 teaspoons of tahini

Morning Snack (school)
- 1 round sesame bread (1st break)
- 1 medium sized fruit (2nd or 3rd break)

Lunch
- 90g roasted fish cooked with 1 teaspoon of olive oil
- 1 medium sized baked potato
- Fresh salad made with raw or boiled vegetables with 1 teaspoon of olive oil

Afternoon Snack
- 1 cup of milk (250ml) 1.5% fat or 1 cup (200g) of yogurt 2% fat
- ½ cup of cereals (whole wheat)

Dinner
- Sandwich with 2 slices of bread (whole wheat) and 30g cottage cheese and vegetables (e.g. tomato, lettuce, cucumber)
- 1 medium sized fruit

Before Bedtime
- 1 cup of milk (250ml) 1.5% fat or 1 cup (200g) of yogurt 2% fat

Day 4th

Breakfast
- 1 cup of milk (250ml) 1.5% fat or 1 cup (200g) of yogurt 2% fat
- ½ cup of cereals (whole wheat)

Morning Snack (school)
- 1 round sesame bread (1st break)
- 1 medium sized fruit (2nd or 3rd break)

Lunch
- 1 cup of lentils cooked with 2 teaspoons of olive oil
- Fresh salad made with raw or boiled vegetables with 1 teaspoon of olive oil
- 1 slice of whole wheat bread

Afternoon Snack
- 1 cup of milk (250ml) 1.5% fat or 1 cup (200g) of yogurt 2% fat
- 1 medium sized fruit

Dinner

Chef's salad:
- 1 egg boiled
- 30g cottage cheese
- 30g turkey boiled chopped
- plenty of vegetables
- 2 grated rusks
- 2 teaspoons of olive oil

Before Bedtime
- 1 cup of milk (250ml) 1.5% fat or 1 cup (200g) of yogurt 2% fat

Diet Meal Plan 3

Day 5th

Breakfast
- 1 cup of milk (250ml) 1.5% fat or 1 cup (200g) of yogurt 2% fat
- ½ cup of cereals (whole wheat)

Morning Snack(school)
- Sandwich with 2 slices of bread (whole wheat) and 30g cottage cheese (low fat) and vegetables (e.g. tomato, lettuce, cucumber) (1st break)
- 1 medium sized fruit (2nd or 3rd break)

Lunch
- 1 cup peas cooked with fresh tomato sauce, 1/2 medium sized potato and 2 teaspoons of olive oil
- Fresh salad made with raw or boiled vegetables with 1 teaspoon olive oil
- 1 medium sized fruit

Afternoon Snack
- 1 cup of milk (250ml) 1.5% fat or 1 cup (200g) of yogurt 2% fat
- 1 medium sized fruit

Dinner
- 1 chicken souvlaki roasted (100g)
- Fresh salad made with raw or boiled vegetables with 1 teaspoon of olive oil
- ½ pita bread whole wheat (30g) without olive oil

Before Bedtime
- 1 cup of milk (250ml) 1.5% fat or 1 cup (200g) of yogurt 2% fat

Day 6th Weekend

Breakfast
- 1 cup of milk (250ml) 1.5% fat or 1 cup (200g) of yogurt 2% fat
- ½ cup of cereals (whole wheat)

Morning Snack
- Sandwich with 2 slices of bread (whole wheat) and 4 teaspoons of tahini
- 1 medium sized fruit

Lunch
- 90g roasted fish cooked with 1 teaspoon of olive oil
- 1/3 cup of rice boiled (brown)
- Fresh salad made with raw or boiled vegetables with 1 teaspoon olive oil

Afternoon Snack
- 1 cup of milk (250ml) 1.5% fat or 1 cup (200g) of yogurt 2% fat
- 1 medium sized fruit

Dinner

Homemade Pizza (ingredients):
- 1 pita bread whole bread(60g)
- vegetables (e.g. tomato, peppers, onions, mushrooms)
- 30g cottage cheese
- 1 slice of turkey boiled chopped

Before Bedtime
- 1 cup of milk (250ml) 1.5% fat or 1 cup (200g) of yogurt 2% fat

Day 7th Weekend

Breakfast
- 1 cup of milk (250ml) 1.5% fat or 1 cup (200g) of yogurt 2% fat
- ½ cup of cereals (whole wheat)

Morning Snack
- Sandwich with 2 slices of bread (whole wheat) and 4 teaspoons of tahini
- 1 medium sized fruit

Lunch
- 90g shrimps cooked with homemade tomato sauce and 1 teaspoon of olive oil
- 1 cup of pasta (whole wheat) cooked
- Fresh salad made with raw or boiled vegetables with 1 teaspoon olive oil

Afternoon Snack
- 1 cup of milk (250ml) 1.5% fat or 1 cup (200g) of yogurt 2% fat
- 1 medium sized fruit

Dinner

Homemade Pizza (ingredients):
- 1 pita bread whole bread(60g)
- vegetables (e.g. tomato, peppers, onions, mushrooms)
- 30g cottage cheese
- 1 slice of turkey boiled chopped

Before Bedtime
- 1 cup of milk (250ml) 1.5% fat or 1 cup (200g) of yogurt 2% fat

Comments

(In the box below please record if you do anything different from what is recommended in your diet meal plan)

Diet Meal Plan 4

**Day 1st**	**Day 2nd**	**Day 3rd**	**Day 4th**
Breakfast<br>• 1 cup of milk (250ml) 1.5% fat or 1 cup (200g) of yogurt 2% fat<br>• ½ cup of cereals (whole wheat)	Breakfast<br>• 1 cup of milk (250ml) 1.5% fat or 1 cup (200g) of yogurt 2% fat<br>• ½ cup of cereals (whole wheat)	Breakfast<br>• 1 cup of milk (250ml) 1.5% fat or 1 cup (200g) of yogurt 2% fat<br>• 2 rusks unsalted (whole wheat) with 2 teaspoons of tahini	Breakfast<br>• 1 cup of milk (250ml) 1.5% fat or 1 cup (200g) of yogurt 2% fat<br>• ½ cup of cereals (whole wheat)
Morning Snack (school)<br>• Sandwich with 2 slices of bread (whole wheat) and 30g cottage cheese and vegetables (e.g. tomato, lettuce, cucumber) (1st break)<br>• 1 medium sized fruit (ideally unpeeled) (2nd or 3rd break)	Morning Snack (school)<br>• Sandwich with 2 slices of bread (whole wheat) and 30g cottage cheese and vegetables (e.g. tomato, lettuce, cucumber) (1st break)<br>• 1 medium sized fruit (ideally unpeeled) (2nd or 3rd break)	Morning Snack (school)<br>• 1 round sesame bread whole wheat (1st break)<br>• 1 medium sized fruit (ideally unpeeled) (2nd or 3rd break)	Morning Snack (school)<br>• 1 round sesame bread whole wheat (1st break)<br>• 1 medium sized fruit (ideally unpeeled) (2nd or 3rd break)
Lunch<br>• 90g roasted chicken's breast without skin, cooked with tomato sauce and herbs<br>• ½ cup of brown rice boiled<br>• Fresh salad made with raw or boiled vegetables, with 2 teaspoons of olive oil	Lunch<br>• 1 cup of pasta (whole wheat) cooked with 1 teaspoon of olive oil<br>• 30g grated cheese (low fat)<br>• Fresh salad made with raw or boiled vegetables with 2 teaspoons of olive oil	Lunch<br>• 90g roasted chicken's breast without skin cooked with lemon and 1 teaspoon of olive oil<br>• 1 medium sized baked potato with its skin<br>• Fresh salad made with raw or boiled vegetables with 2 teaspoons of olive oil	Lunch<br>• 1 cup of lentils cooked with 2 teaspoons of olive oil<br>• Fresh salad made with raw or boiled vegetables with 1 teaspoon of olive oil<br>• 1 slice of whole wheat bread (30g)
Afternoon Snack<br>• 1 cup of milk (250ml) 1.5% fat or 1 cup (200g) of yogurt 2% fat<br>• 1 medium sized fruit (ideally unpeeled)	Afternoon Snack<br>• 1 cup of milk (250ml) 1.5% fat or 1 cup (200g) of yogurt 2% fat<br>• 1 medium sized fruit (ideally unpeeled)	Afternoon Snack<br>• 1 cup of milk (250ml) 1.5% fat or 1 cup (200g) of yogurt 2% fat<br>• ½ cup of cereals (whole wheat)	Afternoon Snack<br>• 1 cup of milk (250ml) 1.5% fat or 1 cup (200g) of yogurt 2% fat<br>• 1 medium sized fruit (ideally unpeeled)
Dinner<br>Greek Dakos salad (ingredients):<br>• 1 medium rusk whole wheat (60g)<br>• 30g cottage cheese<br>• grated or chopped tomato<br>• 2 teaspoons of olive oil<br>• 1 medium sized fruit (ideally unpeeled)	Dinner<br>• Caesar's Salad (ingredients):<br>• lettuce<br>• 30g cottage cheese<br>• 30g roasted chicken's breast without skin chopped<br>• 2 grated rusks unsalted<br>• 2 teaspoons of olive oil<br>• 1 medium sized fruit (ideally unpeeled)	Dinner<br>• Sandwich with 2 slices of bread (whole wheat) and 60g cottage cheese and vegetables (e.g. tomato, lettuce, cucumber)<br>• 1 medium sized fruit (ideally unpeeled)	Dinner<br>Caesar's Salad (ingredients):<br>• 30g cottage cheese<br>• 30g roasted chicken's breast without skin chopped<br>• 2 grated rusks unsalted<br>• 1 teaspoon of olive oil<br>• plenty of vegetables
Before Bedtime<br>• 1 cup of milk (250ml) 1.5% fat or 1 cup (200g) of yogurt 2% fat	Before Bedtime<br>• 1 cup of milk (250ml) 1.5% fat or 1 cup (200g) of yogurt 2% fat	Before Bedtime<br>• 1 cup of milk (250ml) 1.5% fat or 1 cup (200g) of yogurt 2% fat	Before Bedtime<br>• 1 cup of milk (250ml) 1.5% fat or 1 cup (200g) of yogurt 2% fat

Diet Meal Plan 4

Day 5th

Breakfast

- 1 cup of milk (250ml) 1.5% fat or 1 cup (200g) of yogurt 2% fat
- ½ cup of cereals (whole wheat)

Morning Snack(school)

- Sandwich with 2 slices of bread (toasted) (whole wheat) and 30g cottage cheese (low fat) and vegetables (e.g. tomato, lettuce, cucumber) (1st break)
- 1 medium sized fruit (ideally unpeeled) (2nd or 3rd break)

Lunch

- 1 cup of peas cooked with fresh tomato sauce and 2 teaspoons of olive oil
- 1 slice of bread whole wheat (30g)
- Fresh salad made with raw or boiled vegetables with 2 teaspoons olive oil
- 1 medium sized fruit (ideally unpeeled)

Afternoon Snack

- 1 cup of milk (250ml) 1.5% fat or 1 cup (200g) of yogurt 2% fat
- 1 medium sized fruit (ideally unpeeled)

Dinner

- 1 chicken breast souvlaki roasted (100g)
- Fresh salad made with raw or boiled vegetables with 2 teaspoons of olive oil
- ½ pita bread whole wheat (30g) without olive oil

Before Bedtime

- 1 cup of milk (250ml) 1.5% fat or 1 cup (200g) of yogurt 2% fat

Day 6th Weekend

Breakfast

- 1 cup of milk (250ml) 1.5% fat or 1 cup (200g) of yogurt 2% fat
- ½ cup of cereals (whole wheat)

Morning Snack

- Sandwich with 2 slices of bread (whole wheat and unsalted) and 4 teaspoons of tahini and 1 teaspoon of honey
- 1 medium sized fruit (ideally unpeeled)

Lunch

- 90g roasted fish
- 1/3 cup of rice boiled (brown)
- Fresh salad made with raw or boiled vegetables with 2 teaspoons of olive oil

Afternoon Snack

- 1 cup of milk (250ml) 1.5% fat or 1 cup (200g) of yogurt 2% fat
- 1 medium sized fruit (ideally unpeeled)

Dinner

Greek Dakos salad (ingredients):

- 1 medium rusk whole wheat (60g)
- 30g cottage cheese
- grated or chopped tomato
- 1 teaspoon of olive oil

Before Bedtime

- 1 cup of milk (250ml) 1.5% fat or 1 cup (200g) of yogurt 2% fat

Day 7th Weekend

Breakfast

- 1 cup of milk (250ml) 1.5% fat or 1 cup (200g) of yogurt 2% fat
- ½ cup of cereals (whole wheat)

Morning Snack

- Sandwich with 2 slices of bread (whole wheat) and 3 teaspoons of tahini and 1 teaspoon of honey
- 1 medium sized fruit (ideally unpeeled)

Lunch

- 90g shrimps cooked with homemade tomato sauce and 1 teaspoon of olive oil
- 1 cup of pasta (whole wheat) cooked
- 1 tablespoon of grated cheese
- Fresh salad made with raw or boiled vegetables with 2 teaspoons of olive oil

Afternoon Snack

- 1 cup of milk (250ml) 1.5% fat or 1 cup (200g) of yogurt 2% fat
- 1 medium sized fruit (ideally unpeeled)

Dinner

- Sandwich with 2 slices of bread (whole wheat), 60g cottage cheese and vegetables (e.g. tomato, lettuce, cucumber)
- 1 medium sized fruit (ideally unpeeled)

Before Bedtime

- 1 cup of milk (250ml) 1.5% fat or 1 cup (200g) of yogurt 2% fat

Comments

(In the box below please record if you do anything different from what is recommended in your diet meal plan)
